# Development of a New Rapid Isolation Device for Circulating Tumor Cells (CTCs) Using 3D Palladium Filter and Its Application for Genetic Analysis

**DOI:** 10.1371/journal.pone.0088821

**Published:** 2014-02-11

**Authors:** Akiko Yusa, Makoto Toneri, Taisuke Masuda, Seiji Ito, Shuhei Yamamoto, Mina Okochi, Naoto Kondo, Hiroji Iwata, Yasushi Yatabe, Yoshiyuki Ichinosawa, Seichin Kinuta, Eisaku Kondo, Hiroyuki Honda, Fumihito Arai, Hayao Nakanishi

**Affiliations:** 1 Aichi Science and Technology Foundation, Knowledge Hub Aichi, Priority Research Projects, Japan; 2 Department of Gastrointestinal Surgery, Aichi Cancer Center Central Hospital, Japan; 3 Department of Micro-Nano Systems Engineering, Graduate School of Engineering, Nagoya University, Japan; 4 Department of Biotechnogloy, Graduate School of Engineering, Nagoya University, Japan; 5 Department of Breast Oncology, Aichi Cancer Center Central Hospital, Japan; 6 Department of Pathology and Molecular Diagnostics, Aichi Cancer Center Central Hospital, Japan; 7 Optnics Precision Co., Ltd., Japan; 8 Division of Oncological Pathology, Aichi Cancer Center Research Institute, Japan; Osaka University, Japan

## Abstract

Circulating tumor cells (CTCs) in the blood of patients with epithelial malignancies provide a promising and minimally invasive source for early detection of metastasis, monitoring of therapeutic effects and basic research addressing the mechanism of metastasis. In this study, we developed a new filtration-based, sensitive CTC isolation device. This device consists of a 3-dimensional (3D) palladium (Pd) filter with an 8 µm-sized pore in the lower layer and a 30 µm-sized pocket in the upper layer to trap CTCs on a filter micro-fabricated by precise lithography plus electroforming process. This is a simple pump-less device driven by gravity flow and can enrich CTCs from whole blood within 20 min. After on-device staining of CTCs for 30 min, the filter cassette was removed from the device, fixed in a cassette holder and set up on the upright fluorescence microscope. Enumeration and isolation of CTCs for subsequent genetic analysis from the beginning were completed within 1.5 hr and 2 hr, respectively. Cell spike experiments demonstrated that the recovery rate of tumor cells from blood by this Pd filter device was more than 85%. Single living tumor cells were efficiently isolated from these spiked tumor cells by a micromanipulator, and *KRAS* mutation, HER2 gene amplification and overexpression, for example, were successfully detected from such isolated single tumor cells. Sequential analysis of blood from mice bearing metastasis revealed that CTC increased with progression of metastasis. Furthermore, a significant increase in the number of CTCs from the blood of patients with metastatic breast cancer was observed compared with patients without metastasis and healthy volunteers. These results suggest that this new 3D Pd filter-based device would be a useful tool for the rapid, cost effective and sensitive detection, enumeration, isolation and genetic analysis of CTCs from peripheral blood in both preclinical and clinical settings.

## Introduction

Despite decades of efforts, to detect and understand their role, CTCs still remain one of the major challenges of basic metastasis research as well as clinical oncology. CTCs were first quantitatively detected by RT-PCR technique using tumor-specific marker genes [1], [2], but the results were insufficient in terms of sensitivity, specificity and reproducibility due to contamination and illegitimate transcription, as well as the indirect method that could not provide direct evidence of the presence of CTCs in the blood. Recent advances in immunomagnetic and micro-device technology made direct visualization of fixed CTC or live CTC possible [3]. Clinical studies using CTC detection devices such as CellSearch system (Veridex, Raritan, NJ), which was recently approved by the US Food and Drug Administration (FDA), demonstrated that CTCs were prognostic markers for patient survival and useful as surrogate biomarkers for various solid tumors such as metastatic breast [4], [5], [6], colorectal [7], [8], prostate [9], and non–small cell lung cancer (NSCLC) [10]. CTCs were also found to be associated with clinical stage, disease recurrence and disease monitoring before and after treatment [11], [12], [13], [14]. More recently, Harber et al. reported having invented a small CTC chip consisting of antibody-coated micropost using microfluidic technology [15]. To date, many such microfluidic devices have been reported using captured antibody [16]. The most commonly used antibody for CTC enumeration is an antibody to epithelial cell adhesion molecule (EpCAM). However, the use of such an epithelial antigen as a positive selection marker is not always optimal because epithelial cell-specific molecules are not infrequently down-regulated by epithelial mesenchymal transition (EMT) generated during tumor progression [17], [18], [19], [20]. Therefore, an epithelial-specific antibody-dependent selection of CTC may still be insufficient despite improvements with the new EMT-related antibody or the use of antibody cocktails [21].

CTC is difficult to detect and isolate because of its rarity. Its level of concentration, 1∼10^2^ in 7.5 mL of blood, makes efficient enrichment a prerequisite for CTC detection, enumeration and isolation in most cases. Various CTC enrichment methods exploit the intrinsic differences between epithelial-derived CTCs and blood cells [22]. Among these, a potential approach independent of captured antibody is size-dependent selection of CTC using various types of filtration techniques [23], [24], [25]. This is based on the fact that almost all cultured epithelial tumor cells are larger than erythrocytes and leukocytes, except for minor subgroups such as small cell lung cancers (SCLC). Advantages of these size-based methods include rapid and efficient enrichment of almost all CTCs, including cells undergoing EMT, with a low cost [26]. Furthermore, this size-based method can easily isolate a single living CTC, which is somewhat difficult to achieve by an antibody-coated microfluidic device and cell sorter type CTC enrichment device [27]. Isolation of a single CTC in the intact state as much as possible from the whole blood is the most important parameter required for an ideal CTC device, because pure CTC without leukocyte contamination permits accurate genetic analysis of mutation, amplification and gene expression as well as a standard cytopathological diagnosis [16], [28], [29], [30]. Despite advances in filter type devices such as on-chip (on-filter) multimarker immunofluorescence analysis and improved software for automated image analysis, not many filter-type devices capable of isolating living CTCs have been reported to date [24], [25]. This is mainly because almost all filter devices are closed systems in which the filter is assembled inside a housing cassette. One potential solution for this problem is an open-type multifunctional filter device capable of enrichment, spreading and alignment of CTC on the filter, which subsequently can be directly and gently picked up by the manipulator.

In order to understand the fundamental role of CTC in hematogenous metastasis and to use it for clinical applications such as early diagnosis of metastatic disease, companion diagnosis and monitoring therapeutic effects, we developed a new size-selective device in this study that can rapidly enumerate and then efficiently isolate a small number of CTCs in a living or fixed condition from peripheral blood, using a unique size-dependent 3D palladium (Pd) filter device and micromanipulation system. We evaluated its performance for CTC detection in both preclinical and clinical setting. The advantage and disadvantage associated with this device will be discussed.

## Materials and Methods

### Reagents

The following pair of antibodies was used in this study: Alexa Fluor 488-labeled anti-human EpCAM mouse monoclonal antibody (MoAb) (Cell Signaling Technology, Danvers, MA), PE-labeled anti-human CD45 (common leukocyte antigen) mouse MoAb (Miltenyi Biotec, Auburn, CA). In some cases, Zenon Alexa fluor 594 (or 647) (Invitrogen, Eugene, OR) conjugated mouse MoAb against a broad spectrum of human cytokeratin (AE1/AE3, Dako, Copenhagen, Denmark) and Zenon Alexa fluor 488 (Invitrogen, Eugene, OR) conjugated humanized HER2 MoAb (Trastuzumab, Chugai, Tokyo, Japan) were used. Hoechst 33342 (Molecular Probes, Eugene, OR) was used for nuclear staining.

### Patients and Ethics Statement

In this pilot clinical study, 19 breast cancer patients with distant metastasis (M1) to the lung, liver, bone, and brain, most of which received various kind of therapies at Department of Breast Oncology, Aichi Cancer Center Central Hospital, 13 breast cancer patients without distant metastasis (M0) and 12 healthy volunteers as negative control were enrolled. The average ages of cancer patients (M1, M0) and healthy was 58, 54 and 38 years, respectively. Patient blood samples (5 ml) were collected in EDTA-2Na tubes from cubital vein, kept at room temperature and used for examination within 4 hours. This study was approved by the institutional review board of the Aichi Cancer Center and all participants provided written informed consent to participate in the study.

### Animals and Ethics Statement

Seven- to eight-week-old male athymic nude mice of KSN strain were obtained from Shizuoka Laboratory Animal Center (Hamamatsu, Japan) and maintained under specific pathogen-free (SPF) conditions. All animal experiments were performed under the experiment protocol approved by the Ethics Review Committee for Animal Experimentation of the Aichi Cancer Center and met the standard as defined by the UKCCCR guidelines.

### Cell Lines

NCI N-87 cell line, a HER2 gene-amplified human gastric carcinoma cell line, and Capan-1 cells, human pancreatic carcinoma cell line with *KRAS* mutation, was obtained from ATCC Cell Bank. COLM-5 cell line, a poorly-differentiated human colonic cancer cell line, was established in our laboratory [31]. The latter cell line was transfected with the pEGFP-C1 plasmid (CLONTECH Laboratories, Palo Alto, CA) using the FuGENE6 transfection reagent (Roche Diagnostics, Basel, Switzerland). The cell line was maintained in Dulbecco's modified Eagle's medium (DMEM) (Nissui Pharmaceutical Co., Ltd., Tokyo, Japan) containing 10% fetal bovine serum (FBS) (GIBCO, Grand Island, NY) with 100 units/ml penicillin and 100 µg/ml streptomycin sulfate (Sigma-Aldrich, St. Louis, MO) and cultured in a humidified 5% CO_2_ incubator at 37°C.

### Cell Size Measurement of Cultured Tumor Cells and Blood Cells

Diameters of cells were measured using Luna cell counter (Logos Biosystems Inc., Gyunggi-Do, Korea) based on imaging analysis. Human and mouse red and white blood cells were prepared by Ficoll-Hypaque gradient centrifugation. Measurement of cells was carried out after 0.04% trypan blue staining according to the manufacturer's protocol.

### Filtration-based Device for Detection and Isolation of CTC from Blood

**Figure 1:**
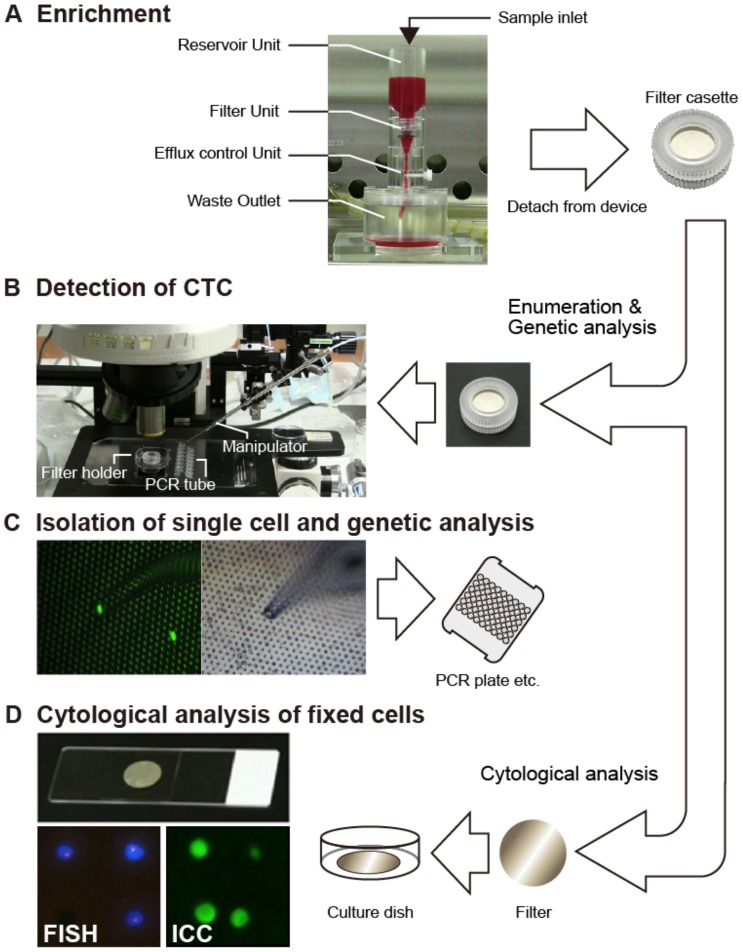
Flowchart of enrichment, enumeration, isolation and molecular analysis of CTC by metal filtration-based device. **A**. Overview of CTC enrichment device which consists of blood reservoir, filter unit and disposal tank. Filter unit (filter cassette) is composed of a palladium (Pd) metal filter placed between upper and lower cassette pieces. Diluted whole blood is applied to the reservoir and filtrated driven by gravity flow without a pump. **B**. After filtration, filter cassette is detached from the device and set up in combination with cassette holder on the upright fluorescence microscope for enumeration and isolation. **C**. Single CTC is isolated with micromanipulation using a glass capillary. Isolated CTC moved into PCR plate and DNA/RNA is extracted and amplified. Mutation and/or gene expression analysis is then performed. **D**. The filter is detached from cassette and is directly stained with immunocytochemistry (ICC) and FISH method.

**Figure 2:**
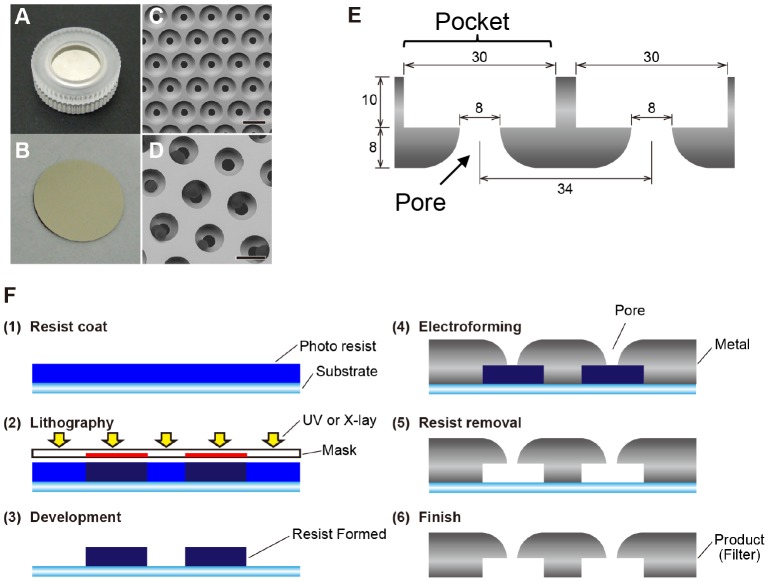
Characterization and production of 3D palladium filter. **A**–**B**. Macroscopic view of filter cassette (A) and the filter (B) sandwiched by the upper and lower cassette piece. **C**–**D**. SEM images of 3D Pd filter (C) and tumor cells trapped in the pockets of the filter (D). Bars = 30 µm. **E**. Schematic of 3D Pd filter, composed of 8 µm-sized pore in lower layer and 30 µm-sized CTC capture hole (pocket) in upper layer with 34 µm pitch. **F**. Basic process of Pd filter production. Process (1–3); Photo-sensitive coating (photo-resist) applied to metal substrate and image transferred by X-ray or UV exposure through photomask develops and rises off solved photo-resist. Process (4–6); Metal molecules (palladium or alloy) electrodeposited to the matrix (substrate) on areas not masked with photo-resist (patterned surface) are thicker than the resist, so as to overhang on the resist formed on the substrate through electroforming. Once the metal is deposited in the desired thickness, the electroformed part can be stripped off from metal substrate and the electroformed product is completed.

Enrichment, isolation and molecular analysis of CTC by filtration-based device were overviewed in figure 1. The CTC enrichment device consists of a blood reservoir, filter unit, efflux control unit and waste outlet. The size of the device is approximately 15 (length) x 13 (width) x 20 (height) cm (Figure 1A). The 3D palladium (Pd) filter is composed of an 8 µm-sized pore in the lower layer and a 30 µm-sized CTC capture hole (pocket) in the upper layer with a 34 µm-pitch (Figure 2E). This Pd filter was placed between the upper and lower filter cassette piece (Figure 2A and 2B). Whole blood obtained from posterior caval vein in mice or cubital veins of patients were 10-fold diluted with PBS and applied to the reservoir directly by gravity flow without a peristaltic pump. After filtration of blood, a filter cassette was fixed on-device with 10% formalin in PBS for 10 min, followed by immersion with 0.5 ml of antibody mixture for 30 min at RT, followed by washing with PBS. These procedures were carried out under a clean condition at least S1000 level. After staining, a filter cassette was detached from the device and plugged into the cassette holder (Chuo SEIKI, Tokyo, Japan) on the stage of the upright fluorescence microscope to fix the cassette and to control the depth of PBS above the filter to the optimal position. The fluorescence-positive cells were then counted and photographed by the pathologist before knowing any clinical data of the patients. Single cell isolation of CTC was carried out with a micromanipulator using a glass capillary (Figure 1B). The isolated CTC or CTC pool was transferred to 0.2 ml of PCR tube or a 96 well PCR plate and genomic DNA or RNA was extracted, amplified, and genetic analysis was performed (Figure 1C). When isolating living CTC, 10% formalin fixation process was omitted.

### Pd Filter Fabrication

3D Pd filter is produced by micro-fabrication technology, consisting of lithography and electroforming processes (Figure 2F). Photo-sensitive coating (photo-resist) is applied to a metal substrate and resist patterns (photomasks) are formed on this conductive substrate by using a UV or X-ray lithography process and rises off solved photo-resist (process 1–3). Metal molecules, Pd or Pd-Nickel (4∶1) alloy, electrodeposited to the matrix (substrate) on areas not masked with photo-resist (patterned surface) were thicker than the resist, so as to overhang on the resist formed on the substrate by electroforming. Once the material is deposited to the desired thickness, the electroformed part can be stripped off from the metal substrate and the electroformed product is completed (process 4–6). The finished filter has holes with a round shape like a bell mouth.

### Fluid Dynamics Simulation in the Filter Device by COMSOL Software

Computational simulation of fluid dynamics such as flow rate and tension or pressure for “like cell” in the 3D metal filter device was carried out in comparison with the 2D metal filter using FEM analysis software (COMSOL Multiphysics 4.0, COMSOL Inc., Sweden) according to the manufacturer's protocol. Filters with or without pocket in the upper layer are referred to as 3D and 2D metal filters, respectively.

### Single Cell (CTC) Isolation from the Filter Cassette

Single cell isolation was carried out on a micromanipulation system (MN-4, MMO-203, Narishige, Tokyo, Japan) mounted upright fluorescence microscope (LV-100ND, Nikon, Tokyo, Japan). Three replicates of a single tumor cell, pools of 5 tumor cells, and pools of 10 tumor cells were isolated with glass micropipettes connected to a pneumatic microinjector (IM-11-2, Narishige). The cells were lysed in a drop of lysis buffer either for RNA or DNA extraction and spun-down into the bottom of 0.2 mL PCR tubes. The cell lysate was then processed to the following steps, or the lysate was stored at −80°C until use. The successful transfer of cells to lysis buffer was verified by direct visualization. Digital images were captured by cooled CCD camera, Ri-1, and NIS elements software (Nikon).

### FISH Analysis

Amplification of the c-erb B-2 gene was determined by a dual-color FISH method using a Passvision HER-2 DNA Probe Kit (Vysis Inc., Downers Grove, IL, USA) according to the manufacturer's protocol. The HER-2/neu –Spectrum Orange probe contains a DNA sequence specific for the c-erb B-2 human gene locus and hybridizes to region 17q11.2-q12 of the human chromosome. The CEP 17 green probe that hybridizes to the D17Z1 locus (centromere region of chromosome 17) was used as a control. The nucleus was counterstained with 4′, 6-diamidino-2-phenylindole (DAPI). The slides were observed under a BX60 fluorescence microscope equipped with a digital camera (DP50, Olympus, Tokyo, Japan). A cell was considered to show amplification when a more than 4 definite orange signals for HER2 was present.

### Amplification of Total RNA and Quantitative RT-PCR

Total RNA was amplified from each sample using Ambion® Single Cell-to-CT Amplification Kit (Life Technologies, Carlsbad, CA) according to the manufacturer's procedures. The gene-specific primers used for pre-amplification are shown in Table S1. Amplified cDNA from each sample was used for quantitative RT-PCR of 4 genes. We measured relative gene expression in triplicate by a LightCycler instrument (Roche Applied Science, Mannheim, Germany) using 10-fold dilution of pre-amplification amplicon as template, each primer, TaqMan probe and TaqMan® Master mix (Roche) in a reaction volume of 20 uL. TaqMan probes (Roche) used here are shown in Table S1 as UPL Number. Glyceraldehyde-3-phosphate dehydrogenase (GAPDH) was used as internal control.

### Amplification of Genomic DNA and Detection *KRAS* Mutation

Genomic DNA was extracted from COLM-5 cells and Capan-1 cells as a wild type and a positive control cells for *KRAS* mutation, respectively, and was amplified by whole genome amplification method using GenomePlex Single Cell WGA Kit (Sigma-aldrich) following the manufacturer's protocol. Amplified products were purified using MinElute Reaction Cleanup Kit (Qiagen, Hilden, Germany) and stored at −20°C until subsequent use. The DNA fragment including exon 2 of the *KRAS* gene was amplified with primers using KOD-plus-neo DNA polymerase (TOYOBO, Osaka, Japan). After amplification, PCR products were purified with MinElute Reaction Cleanup Kit and purified amplicon was subjected to cycle sequence using the BigDye Terminator Cycle Sequencing Kit v 3.1(Life Technologies). To remove unincorporated dye terminators, sequencing reaction products were applied to DyeEx2.0 Spin Kit (Qiagen). Then the products were subjected to ABI PRISM 3100 (Life Technologies). Both the forward and reverse sequences obtained were analyzed with BLAST and by manual review.

### Detection of Metastasis in Mice by GFP Fluorescence Imaging

In the spontaneous metastasis model, exponentially growing EGFP-tagged COLM-5 cells were harvested with trypsin/EDTA, washed with Hank's balanced salt solution (HBSS) and re-suspended in HBSS. A tumor cell suspension (5×10^6^/0.2 ml) was subcutaneously injected into the back and lower abdominal flanks of nude mice. One ∼3 months later, blood was harvested from the posterior caval vein of the mice and the lung, liver and kidney were removed. CTC and metastasis with GFP fluorescence were detected by a fluorescent microscope as described previously [32]. This system consists of a LG-PS halogen source that produces blue light (excitation: 450–490 nm) and a dissecting microscope with a cut filter (emission: 530 nm) (SZ40-GFP, Olympus, Tokyo) [33]. GFP images were captured on a Windows PC with Nikon Capture 3 software.

### Statistical Analysis

The statistical significance of differences in data between groups was determined by applying Student's *t*-test or Welch's two-tailed *t*-test.

## Results

### Cell Size Variation of Cancer Cells and Normal Blood Cells

Eleven varieties of cultured tumor cells of human origin including small cell lung cancer cell line and of mouse origin were measured along with red/white blood cells to compare their actual cell size. Although cells of human origin tend to be larger than cells of mouse origin, cell size distribution of various cancer cell lines ranged from 10 to 20 µm, and erythrocytes/leukocytes ranged from 6 to 8 µm, indicating that 8 µm is the optimal size for separating cancer cells from human blood cells (Figure S1).

### Overview of CTC Enrichment and Isolation Procedure

Workflow of enrichment, isolation and molecular analysis of CTC by filtration-based device is overviewed in figure 1. Filtration of blood and subsequent on-filter fixation/staining of the enriched CTCs with Alexa488-EpCAM and PE-CD45 antibody mixture or other labeled antibodies such as HER2 and ER/PgR in cases of breast cancer took 20 min and 45 min, respectively (Figure 1A). Enumeration of CTC was performed by setting the filter cassette detached from the device onto the cassette holder on the upright fluorescence microscopy using a 10× or 20× objective lens. Fluorescent microscopic detection and identification of CTC was carried out through scanning the entire field of the filter with three fluorescence wavelengths (green, red and blue) by the pathologist. This enumeration step, including saving images, took less than 20 min. Therefore, the total time taken for enumeration of CTC from blood filtration to finishing CTC counting is within 1.5 hr (Figure 1B). Single CTC isolation was then performed on the upright fluorescence microscope by direct picking a CTC trapped in the pocket of the upper layer of the filter, using a glass capillary-type manipulator with green fluorescence as a guide for CTC. The resultant isolated CTCs were then transferred to a PCR tube or plate for subsequent genetic analysis (Figure 1C). Furthermore, FISH staining of HER2 in breast cancer cases was successfully performed on filter. Hard treatments such as denaturation with 1N HCl and hybridization at 75°C during FISH staining had no harmful effect on the filter (Figure 1D).

### Production and Characterization of 3D Metal Filter

The filter unit is composed of a filter cassette in which a Pd filter is sandwiched between the upper and lower ring (Figure 2A and 2B). This Pd filter is a 3D filter that consists of two layers, an 8 µm-sized pore in the lower layer and a 30 (or 24) μm-sized capture hole (pocket) in the upper layer (Figures 2C–2E). The 3D filter was made by micro-fabrication technology, consisting of the lithography and electroforming processes (Figure 2F). The resultant filter is an extremely precise filter with a highly homogenous, accurate size of pore (8±0.5 µm) and pocket (30±0.5 µm), sharp edges, and high pore density (100,000 pores/cm^2^) (Figure 2C). This filter can catch and align living CTCs in the pocket on the filter (Figure 2D). Furthermore, this filter, which was electroformed by pure Pd or a Pd alloy (Pd: Nickel = 4∶1), showed less toxicity to cultured cells during 3 days of culture than a pure Nickel filter, which instead showed substantial toxicity to the cultured cells (data not shown). This indicated the biochemically safe properties of the 3D Pd filter.

### Simulations of Fluid Dynamics in the Filter Device by COMSOL

**Figure 3:**
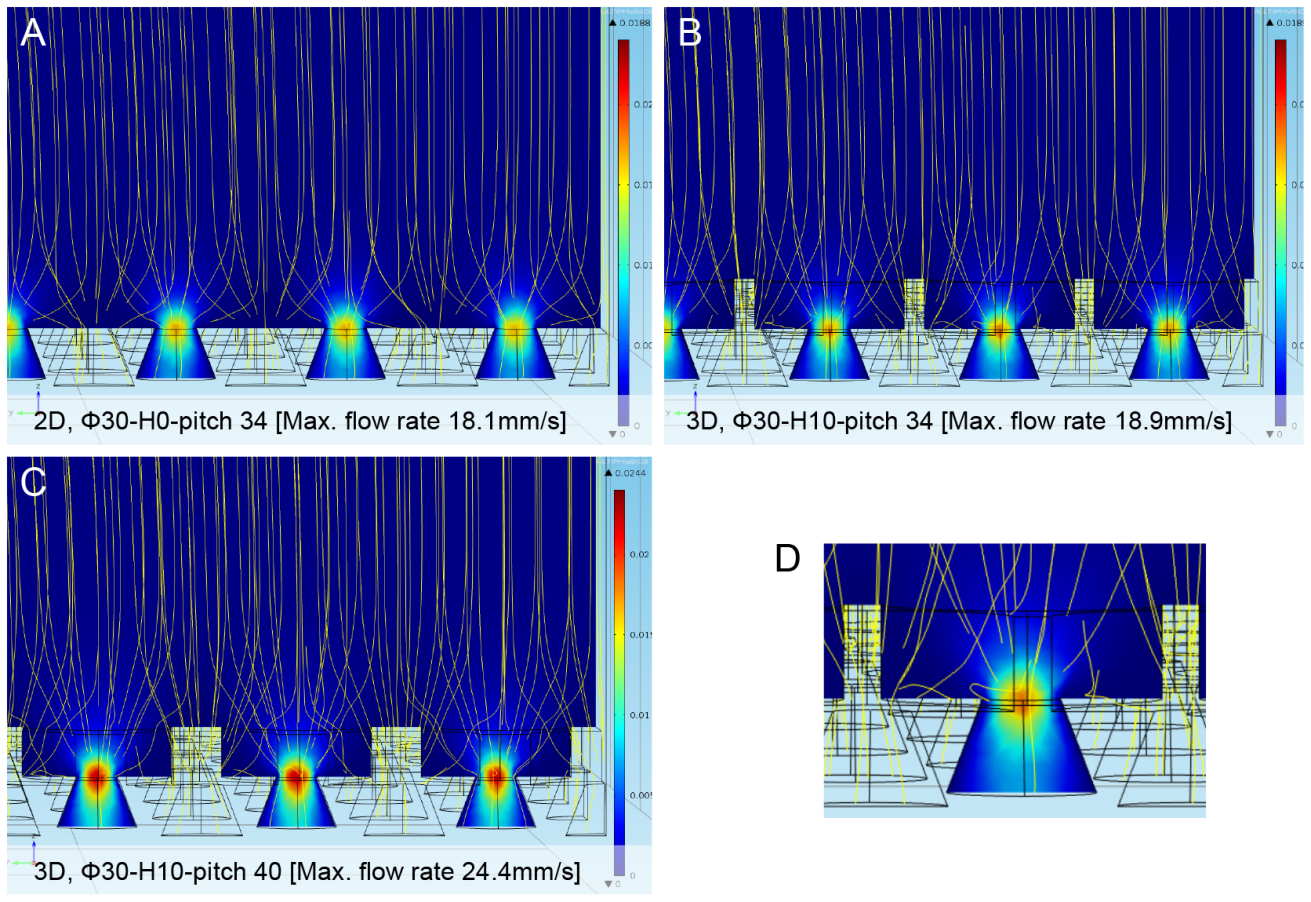
Computational simulations of fluid dynamics of 2D and 3D Pd filter. Fluid dynamics of the 2D and 3D metal filter devices were analyzed using FEM analysis software as described in the Materials and Methods. **A**–**B**. Comparison of fluid field simulation between high pore density (pitch 34 µm) 2D (A) and 3D Pd filter devices (B) without cells showing velocity and streamline (yellow) around the area of capturing pores. **C**. Fluid field simulation of 3D Pd filter device with low pore density (pitch 40 µm). Pseudo color image of the flow rate around the pore as shown in the right corner reveals higher flow rate in the low pore density filter. **D**. Enlarged view of 3D Pd filter devices shown in B. Each calculated values are described in the bottom of each panel. Parameters such as φ, H and pitch shown in figures are follows; φ = pocket diameter, H = height of the pocket, Pitch = distance between pore.

COMSOL analysis showed that when constant velocity (0.3 mm/s) was applied, the maximum flow velocity around the constricted part of the filter (pore) was slower in the high density 3D φ30-H10-pitch34 filter (18.9 mm/s, 100,000 pores/cm^2^) than in the low density 3D φ30-H10-pitch40 filter (24.4 mm/s, 40,000 pores/cm^2^) (Figure 3B and 3C), but there was no significant difference in velocity between the 2D φ30-H10-pitch34 (18.1 mm/s) and 3D φ30-H10-pitch34 filter (18.9 mm/s) (Figure 3A and 3B). This indicated that the pore number is critical for determining the flow rate of the filter, and that the higher the pore number, the lower the flow rate in the filter device. Furthermore, as expected, structures of filters such as 2D and 3D did not significantly affect the flow rate of the filter device. A similar inverse correlation was obtained between the pressure of cancer cells trapped in the filter pore and the pore number. Tension on a plugged-like cell for 2D Pd filter device with low (pitch 50 µm) and high (pitch 30 µm) pore density was calculated as 45 pascal and 18 pascal, respectively, in the case of constant flow rate of 2.5 ml/min (data not shown). Interestingly, the streamline around the capturing pore showed that recirculation and an eddy appeared in the margin of the pocket in 3D filters with φ30-H10-pitch34 parameter (Figure 3D), but not in 2D filters, suggesting that a local turbulent-like flow in the 3D filter device may be present.

### Recovery Rate of Spiked Cancer Cells in Blood by the Filter Device

**Figure 4:**
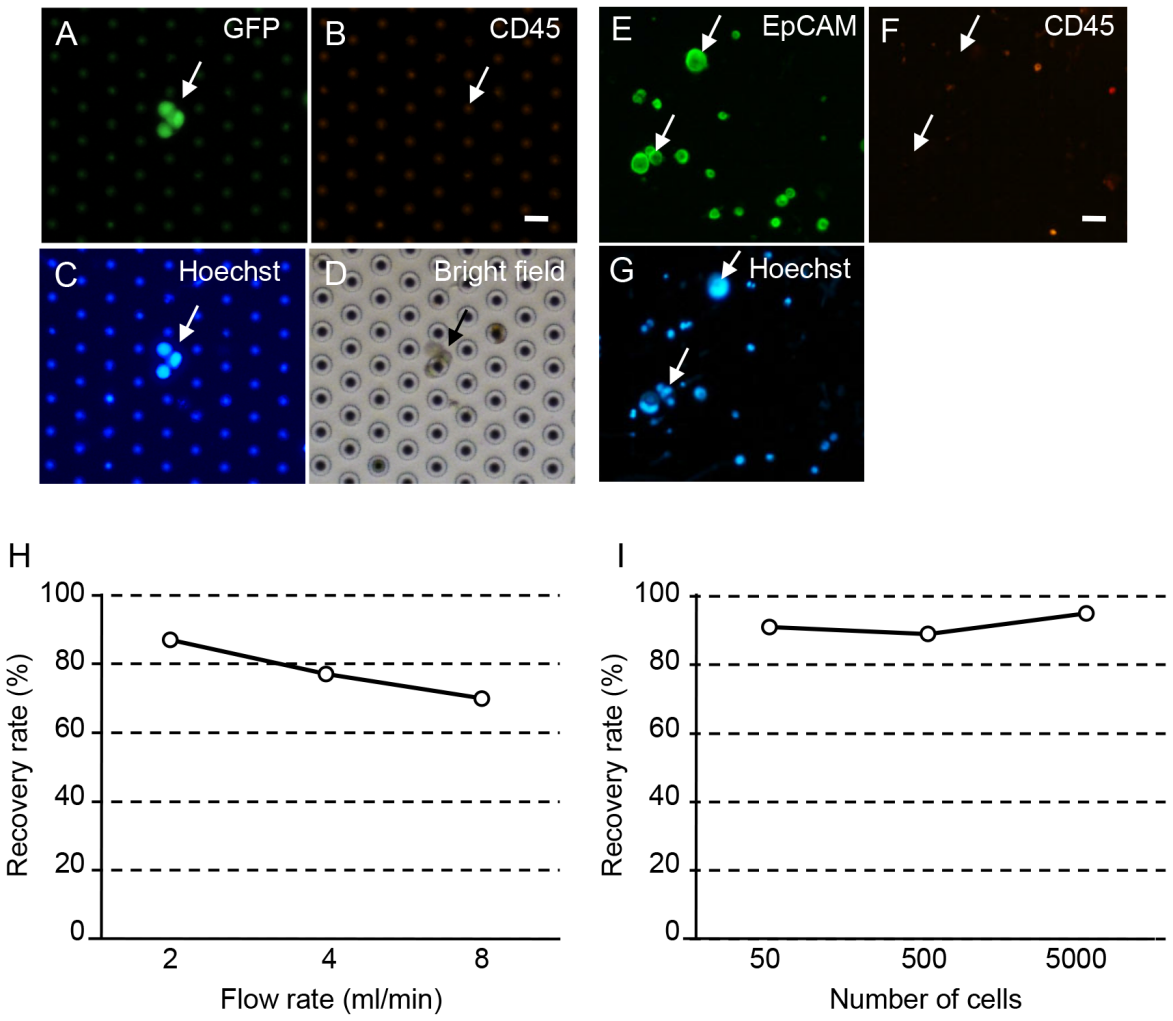
Recovery rate of the spiked cancer cells by 3D Pd filter device. **A**–**D**. Representative recovered spiked GFP-tagged colonic cancer cells (arrows) showing GFP+/CD45−/Hoechst33342+ phenotypes after filtration of 5000 COLM5-EGFP cells spiked into 2 ml normal blood. Images of GFP (A), PE-CD45 (B), Hoechst33342 (C) and bright field (D) are shown. Bars = 40 µm **E**–**G**. Representative recovered spiked GFP non-tagged gastric cancer cells (arrows) showing EpCAM+/CD45−/Hoechst33342+ phenotypes after filtration of 5000 NCI N-87 cells spiked into 2 ml of blood. Images of Alexa488-EpCAM (F), PE-CD45 (G) and Hoechst33342 (H) are shown. Bars = 40 µm **H**. Recovery rate of spiked tumor cells according to flow rate of the filter device. **I**. Recovery rate of spiked tumor cells according to the number of tumor cells spiked into blood.

On the basis of the above-described study, we used a 3D φ30-H10-pitch34 Pd (or Pd alloy) filter with maximal pore density (100,000/cm^2^) as the best filter for rapid, gentle enrichment, and subsequent isolation of living CTC. In a spike experiment, two staining methods were used for detection of spiked epithelial tumor cells in the blood. In the spike experiment using GFP-tagged COLM-5 tumor cells, GFP+/CD45−/Hoechst33342+ cells were recognized as tumor cells (Figures 4A–4D). In the spike experiment using GFP non-tagged tumor cells (NCI N-87), cells with EpCAM+/CD45−/Hoechst33342+ and EpCAM−/CD45+/Hoechst33342+ were recognized as tumor cells and leukocytes, respectively (Figures 4E–4G).

Using this spike experiment system, we evaluated the recovery rate of this device at various flow velocities ranging from 2 to 8 ml/min. We found that the recovery rate decreased with the increased flow rate, and that a 2.0–2.5 ml/min flow rate produced the best results in which the recovery rate was approximately 90% and the treatment time taken for filtration of 5 ml blood was about 20 min (Figure 4H). Furthermore, the recovery rate of COLM5-EGFP cells (or NCI N-87 cells) by this CTC isolating device after spiking of 50, 500 and 5000 cells into 2.5 ml of blood was more than 90% (Figure 4I). In addition, in spike experiments with a small cell number such as 10 cells, more than 70% of cells were recovered by this device (data not shown).

### Detection and Enumeration of CTC from Xenografted Tumor Model in Mice

**Figure 5:**
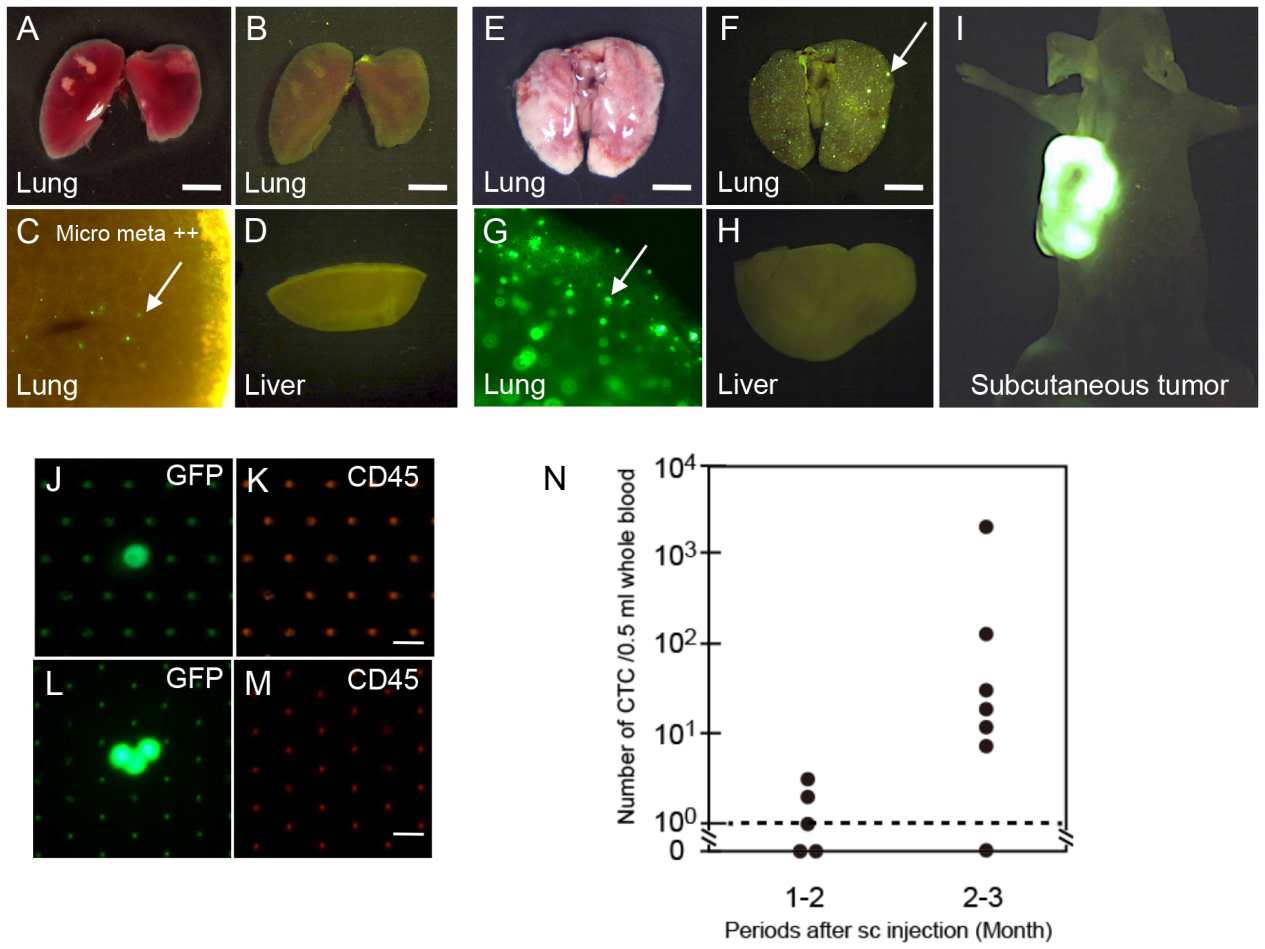
Detection of CTCs from blood in the spontaneous metastasis model of nude mice after sc injection of GFP-tagged COLM-5 human colon cancer cells. **A**–**I**. Sequential observation of lung and liver metastasis and detection of CTC in the blood of nude mice (arrows indicate lung metastasis). **A**–**D**. Micrometastasis in the lung 1–2 months post-injection (A, B. bright and dark field image of the lung, C. Fluorescence microscopic view of the lung, D. Liver). Bar = 3 mm. **E**–**I**. Macroscopic and microscopic metastasis in the lung 2–3 months post-injection (E, F. bright and dark field image of the Lung, G. Fluorescence microscopic view of the lung, H. Liver, I. Subcutaneous tumor). Bar = 3 mm. **J**–**K**. Representative single CTC in the blood from mice 1–2 months post-injection (n = 5). **L**–**M**. Representative CTC cluster in the blood from mice 2–3 months post-injection (n = 7). Bar = 30 µm. **N**. Changes in CTC number in the blood with time.

To evaluate the performance (sensitivity and specificity) of this 3D Pd filter device in metastatic disease *in vivo*, we developed mouse CTC models as reported previously [34], [35] and tested the diagnostic potential of this device. GFP-expressing metastatic colonic cancer cells (COLM5-EGFP) were subcutaneously (sc) injected, and 2–3 months later, they metastasized to the lung spontaneously. Lung metastasis at the micrometastasis level was observed 1–2 months after sc injection of metastatic tumor cells (Figures 5A–5D) and macroscopic lung metastasis was observed 2–3 months post sc injection (Figures 5E–5I). Using this metastasis model, we tested whether or not CTC can be detected by this device. In this spontaneous mouse metastasis model, we could reproducibly detect a significant number of CTCs (10–1000<) in the blood of mice 2–3 months after sc injection (Figure 5L and 5M), but not in mice 1–2 months after injection (Figure 5J and 5K). The number of CTCs clearly increased with progression of lung metastasis (Figure 5N), but the lung metastasis still remained small at less than 1 mm in diameter in this CTC model (Figure 5F), indicating that CTC appears at a relatively early stage of metastatic development in mice.

### Detection and Enumeration of CTC from Clinical Samples

**Figure 6:**
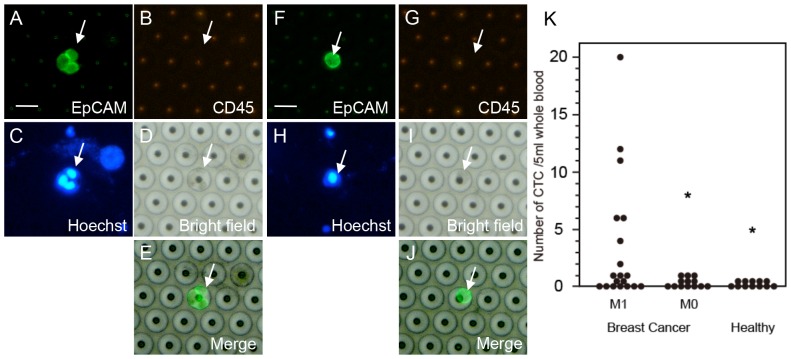
Detection and enumeration of CTC from patients with breast cancers. **A**–**E**. Representative CTC cluster showing EpCAM+/CD45−/Hoechst33342+ pattern. **F**–**J**. Representative single CTC with EpCAM+/CD45−/Hoechst33342+ pattern. Arrows indicate CTC. **K**. Quantitative comparison of CTC number in blood from 19 patients with metastatic breast cancer (M1) and blood from 13 patients without metastasis (M0) and 12 healthy volunteers. Note that CTC was detected in 3 out of 13 M0 patients, but not detected at all in healthy volunteers. (A, F: Alexa488-EpCAM, B, G: PE-CD45, C, H: Hoechst33342, D, I: bright field, E, J: Merge image between EpCAM and bright field. *P<0.05 (vs M1), Bars = 30 µm.

To evaluate usability of the filter device for detecting CTC in the clinical setting, we next examined CTC from the blood of 9 breast cancer patients and 5 healthy volunteers. CTC was identified based on the EpCAM+/CD45−/Hoechst33342+ staining pattern and was observed in a small cluster pattern (Figures 6A–6E) and as a single cell (Figures 6F–6J). In some cases, we confirmed CTC with EpCAM+/cytokeratin (AE1/AE3)+/Hoechst33342+ expression patterns (data not shown). “Events” showing EpCAM+/CD45+/Hoechst33342+ pattern and EpCAM−/CD45−/Hoechst33342+ pattern were judged as unknown cells and artificial substances, respectively and eliminated from CTC [20]. On the bright field, cells captured in the filter pocket were visible, but the image was not always clear like polymer beads. Although the present clinical study is still small in sample-size, a significant increase in CTC numbers (P<0.05) was observed from the blood of patients with metastatic breast cancers (M1, n = 19) (average CTC number = 3.37), compared with patients without metastasis (M0, n = 13) (average CTC number = 0.23) and healthy volunteers (n = 12) (average CTC number = 0) (Figure 6K), suggesting promising clinical usefulness of this device.

### Genetic Analysis of Tumor Cells Isolated from Blood by the Filter Device

**Figure 7:**
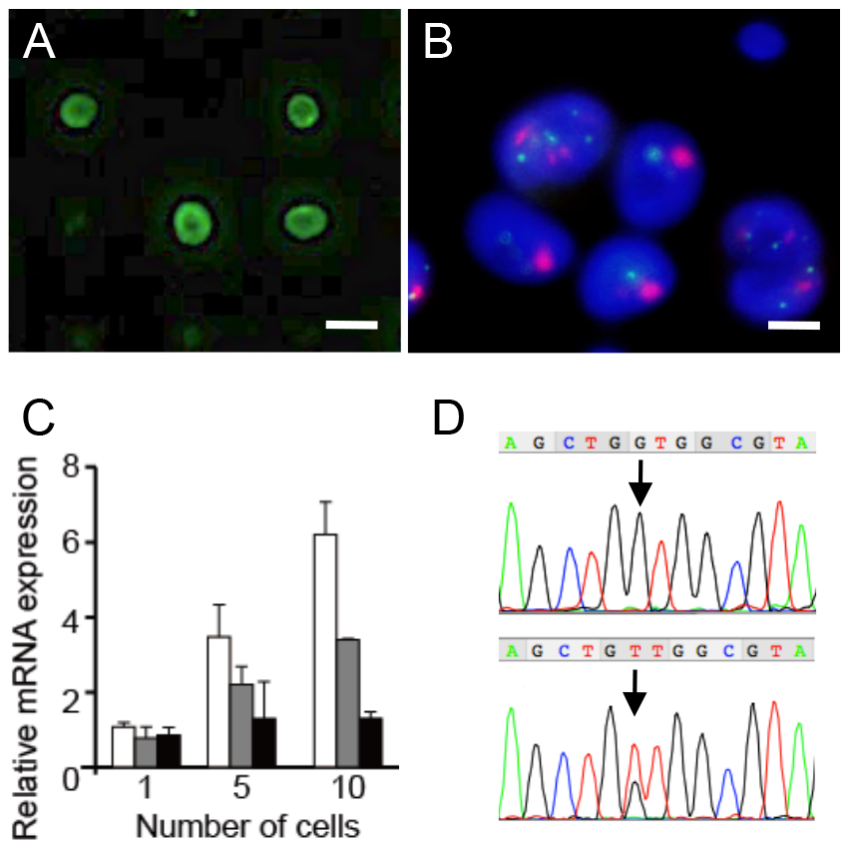
Genetic analysis of isolated spiked tumor cells in blood by 3D Pd filter device. **A**. Immunofluorescence analysis of HER2-positive gastric cancer cells (NCI N-87) on filter. Bar = 30 µm. **B**. Dual-color FISH analysis of NCI N-87 cells on filter. Cluster pattern HER2 gene amplification (red) and 2 copies of centromere signal (green) were observed in the cells. Bar = 30 µm. **C**. qRT-PCR analysis of single, five and ten NCI N-87 cells isolated by the filter device. White box = *HER2*, Gray box = E-cadherin, Black box = *EGFR*. Bars = SD. **D**–**E**. *KRAS* (exon 2) mutation analysis of isolated single mutated Capan-1 pancreatic cancer cell (D) and wild type COLM-5 colon cancer cell (E) by direct sequencing. Arrow indicates mutated base.

The CTC isolation device can be used as a multifunctional device. One of its uses is for on-filter IHC analysis of HER2 and ER/PgR protein expression and FISH analysis of HER2 gene amplification in breast cancer cases, in addition to routine EpCAM and CD45 staining (Figure 1D and Figure 7A and 7B). Another use is for genetic analysis including gene expression analysis of tumor biomarkers such as HER2/E-cadherin by qRT-PCR, and mutation analysis of oncogenes/suppressor oncogenes such as *EGFR/KRAS* by PCR-based whole genome amplification (WGA) and subsequent direct sequencing (Figures 7C–7E). Wild type COLM-5 cells and *KRAS* codon 12 mutated Capan-1 cells (each 500 cells) were spiked into normal healthy blood (2 ml) and resultant isolated spiked tumor cells by 3D filter device were successfully analyzed for *KRAS* mutation. *KRAS* wild type status was confirmed in 3 out of 3 single cells isolated from spiked COLM-5 cells. *KRAS* codon 12 mutation (GGT→GTT) could be detected in 3 out of 4 single cells isolated from spiked Capan-1 cells (Figure 7D and 7E). On the other hand, single cell RT-PCR analysis of mRNA expression of isolated spiked HER2-positive NCI N-87 cells showed that HER2 gene expression increased in cell number-dependent manner, but expression of gene such as E-cadherin and EGFR without gene amplification, especially the latter, could not be detected in a cell number-dependent manner (Figure 7C), suggesting lower reproducibility of gene expression analysis at a single cell level in some low expressing genes than mutation analysis.

## Discussion

In the present study, we developed a new filtration-based CTC isolation device which consists of three major components: a 3D Pd filter device including a filter cassette and a cassette holder that connect the filter device and fluorescence microscopy, and a micromanipulator for single cell isolation. This filtration-based single CTC isolation device has the following advantages.

The filter used in this study is a 3D palladium filter, with a double-layered structure consisting of a pocket in the upper layer to trap a single CTC, and 8-μm pores to wash out blood cells in the lower layer. This 3D Pd filter is fabricated by high-precision lithography with electroforming technology. A 3D Pd filter like this with high pore density (100,000/cm^2^) and a wide pocket (30 µm) supported by a narrow (width; 10 µm) metal frame is difficult to produce by other metal-forming processes such as laser cutting, photo-etching, stamping and machining. Our litho-electroformed Pd filter has high resolution and superior properties over basic metal, allowing it to form a complex 3D filter with fine and uniform geometries and edge definition, as shown in figure 2. Consequently, filtration and staining from whole blood without hemolysis is completed within 1 hr, and the recovery rate of spiked cancer cells by this Pd filter device is sufficiently high (more than 85%) for human breast and gastrointestinal cancer cell lines, with a cell size ranging from 12∼17 µm in diameter. The recovery rate of this 3D Pd filter device is equal or superior to other filter-based methods as reported previously [36], [37], [38]. The Pd filter is autoclavable and resistant to acid and sodium hypochlorite treatment. Therefore, the litho-electroforming Pd filter also has tight tolerances, high repeatability and reusability. The new filter is also relatively low cost, compared with other filters that are micro-fabricated by silicon and parylene [36], [37]. Even in the case with Pd∶Nickel = 4∶1 alloy, the filter is less toxic than other metal filters made of pure nickel, indicating its biochemically safe properties. As for the filter-based CTC detection device, several investigators have reported rapid and low-cost CTC enrichment using a polycarbonate (PC) filter [23], [25]. These PC filters are cost-effective and practically useful, but are produced by track etching, which generates pores at random locations. As a result, there are some problems with relatively low capture efficiency due to pore fusion and low pore density [38]. More recently, microfabricated metal filters and 3D filter devices for CTC enumeration have been reported to be useful [24], [37], but they are specialized for enumeration and on-filter immunohistochemical and FISH analysis of CTC. To our knowledge, ours is the first 3D Pd filter device that has dual capacity to rapidly enumerate the CTC number after on-device staining of the filter for EpCAM, CD45 and Hoechst33342 and to align single CTC in the individual pocket in the upper layer, so as to isolate live single CTCs by the manipulator for subsequent single-cell genetic analysis.

A further advantage of this new 3D Pd filter device is its potential for rapid and gentle isolation of single live CTC with minimum cellular stress and subsequent genetic analysis. The high pore density and 3D structure of our filter allows a relatively low flow rate at the pore site and the turbulent-like flow in the pocket of the filter, respectively, resulting in reduced shearing force on the CTC as revealed by COMSOL analysis (Figure 3). Such CTCs caught in the 8 µm pore can be gently released by the weak reverse-pressure with a syringe pump or low-speed centrifugation if necessary. These CTCs labeled with fluorescence, are located within the individual pocket of the upper layer of the filter, and therefore, can be selectively aspirated by a micromanipulator. Residual leukocytes and erythrocytes present in other pockets were not contaminated in this process. To date, there have been only a few reports on filtration-based device capable of isolation of CTC. CTC isolation on the polycarbonate (PC) filter sometimes requires micro-dissection of membrane or pretreatment to remove blood cell clumps on the PC filter for micromanipulation. Tumor cells on the 3D Pd filter device, on the other hand, can be directly isolated by the manipulator without such pretreatments.

Genetic analyses such as mutation and gene expression analysis of isolated tumor cells in spike experiments showed that *KRAS* mutation of spiked tumor cells was reproducibly detected at a single cell level, whereas gene expression at a relatively low level such as EGFR without gene amplification was difficult to detect quantitatively at a single cell level, indicating lower reproducibility of single cell gene expression analysis than single cell mutation analysis except for HER2 expression in the HER2 gene-amplified cells [39], [40]. At present, to reliably quantitate a low level of multiple gene expression, more than 5 cells are needed. Further study is warranted to develop more sensitive gene expression analysis like a single-cell microfluidics-based RT-PCR analysis [19].

Still another key finding of the present study is that the CTC detection efficacy of 3D Pd filter device was validated by two types of *in vivo* models such as mouse CTC model and clinical samples in addition to the *in vitro* spike experiment. In fact, we demonstrated that a significant increase in CTC numbers and positivity rate was observed from the blood of patients with metastatic breast cancer (M1), compared with patients without metastasis (M0) and healthy volunteers. To confirm that CTCs reflect metastatic progression, we sequentially observed CTCs in mice bearing lung metastasis. The results clearly showed that CTCs were successfully detected from blood of mice bearing spontaneous lung metastasis, and the CTC number increased with progression of lung metastasis in mice, indicating the significance of CTC enumeration for early detection of metastasis as reported previously [41], [42].

The disadvantage with this metal filter device is the need to use upright fluorescence microscopy rather than the usual inverted fluorescence microscopy due to nontransparency of the metal filter. However, this can be virtually overcome by adding an upright halogen light source for bright field observation and the use of a long-distance objective lens for manipulation of CTC. Another problem with this new filter device is that CTC handling such as aspiration and ejection of single cells into the PCR tube by the micromanipulator is still time-consuming (5∼10 min/cell). One way to overcome this is the development of rapid, automated manipulation by robotics, which is now under investigation in our laboratories [22]. Furthermore, there is also some potential limitation with our 3D filter device with 8-μm pore size including the risk of losing CTCs undergoing EMT, small CTC and CTC from patients with small cell lung cancer. Further study is needed to address this issue by the introduction of a 3D filter with a 7-μm pore diameter.

In conclusion, we developed a new filtration-based CTC isolation device and evaluated its performance both *in vitro* and *in vivo*. Our results definitely show that this new 3D Pd filter device is a rapid, convenient, reliable and highly sensitive device with low cost, and is therefore a very promising tool for the detection, enumeration, isolation and genetic analysis of CTCs both in preclinical and clinical settings.

## Supporting Information

Figure S1 **Cell size variation of cancer cells and normal blood cells of human and mouse origin.** **A**. Representative results of image-based cell size analyzer. SCLC, human small cell lung cancer cell line, Ca9-22, human tongue cancer cell line, COLM-5, human colon cancer cell line. Inset displays image of analyzed cells. **B**. Results of human blood cells, RBC, red blood cell, WBC, white blood cell. **C**. Measurements of cell diameter in 6 human and 5 mouse cancer cell lines. Red and blue symbol shows cancer cell lines and blood cells, respectively.
(PPTX)

Table S1 **Primer and Probe information.**
(XLSX)
